# 6-Bromo-1-methyl-4-[2-(1-phenyl­ethyl­idene)hydrazinyl­idene]-3,4-dihydro-1*H*-2λ^6^,1-benzothia­zine-2,2-dione

**DOI:** 10.1107/S1600536812051380

**Published:** 2013-01-04

**Authors:** Muhammad Shafiq, M. Nawaz Tahir, William T. A. Harrison, Iftikhar Hussain Bukhari, Islam Ullah Khan

**Affiliations:** a Department of Chemistry, Government College University, Faisalabad 38000, Pakistan; b Department of Physics, University of Sargodha, Sargodha, Pakistan; c Department of Chemistry, University of Aberdeen, Meston Walk, Aberdeen AB24 3UE, Scotland; d Materials Chemistry Laboratory, Department of Chemistry, Government College University, Lahore, Pakistan

## Abstract

In the title compound, C_17_H_16_BrN_3_O_2_S, the dihedral angle between the aromatic rings is 1.24 (15)° and the C=N—N=C torsion angle is 167.7 (3)°. The conformation of the thia­zine ring is an envelope, with the S atom displaced by 0.805 (3) Å from the mean plane of the other five atoms (r.m.s. deviation = 0.027 Å). In the crystal, C—H⋯O inter­actions link the mol­ecules into *C*(10) [010] chains. A weak C—H⋯π inter­action is also observed.

## Related literature

For the synthesis and biological activity of the title compound and related materials, see: Shafiq, Zia-Ur-Rehman *et al.* (2011 ▶). For further synthetic details, see: Shafiq, Khan *et al.* (2011 ▶).
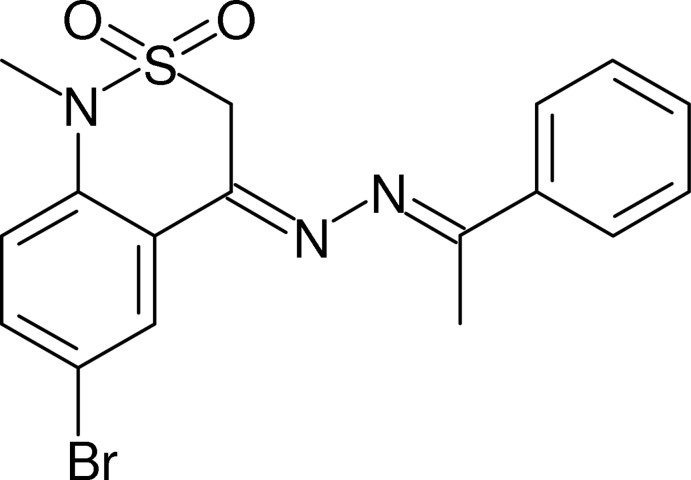


## Experimental

### 

#### Crystal data

C_17_H_16_BrN_3_O_2_S
*M* *_r_* = 406.30
Monoclinic, 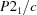

*a* = 16.4369 (13) Å
*b* = 6.5400 (5) Å
*c* = 16.5025 (17) Å
β = 104.312 (4)°
*V* = 1718.9 (3) Å^3^
*Z* = 4
Mo *K*α radiation
μ = 2.53 mm^−1^
*T* = 296 K
0.34 × 0.22 × 0.20 mm

#### Data collection

Bruker APEXII CCD diffractometer
Absorption correction: multi-scan (*SADABS*; Bruker, 2007 ▶) *T*_min_ = 0.518, *T*_max_ = 0.603
7358 measured reflections
3213 independent reflections
2256 reflections with *I* > 2σ(*I*)
*R*_int_ = 0.026

#### Refinement

*R*[*F* ^2^ > 2σ(*F* ^2^)] = 0.037
*wR*(*F* ^2^) = 0.082
*S* = 1.02
3213 reflections
219 parameters
H-atom parameters constrained
Δρ_max_ = 0.56 e Å^−3^
Δρ_min_ = −0.51 e Å^−3^

### 

Data collection: *APEX2* (Bruker, 2007 ▶); cell refinement: *SAINT* (Bruker, 2007 ▶); data reduction: *SAINT*; program(s) used to solve structure: *SHELXS97* (Sheldrick, 2008 ▶); program(s) used to refine structure: *SHELXL97* (Sheldrick, 2008 ▶); molecular graphics: *ORTEP-3* (Farrugia, 1997 ▶); software used to prepare material for publication: *SHELXL97*.

## Supplementary Material

Crystal structure: contains datablock(s) global, I. DOI: 10.1107/S1600536812051380/ng5314sup1.cif

Structure factors: contains datablock(s) I. DOI: 10.1107/S1600536812051380/ng5314Isup2.hkl

Supplementary material file. DOI: 10.1107/S1600536812051380/ng5314Isup3.cml

Additional supplementary materials:  crystallographic information; 3D view; checkCIF report

## Figures and Tables

**Table 1:** Hydrogen-bond geometry (Å, °) *Cg*2 is the centroid of the C1–C6 ring.

*D*—H⋯*A*	*D*—H	H⋯*A*	*D*⋯*A*	*D*—H⋯*A*
C2—H2⋯O2^i^	0.93	2.49	3.280 (4)	143
C13—H13⋯*Cg*2^ii^	0.93	2.65	3.445 (3)	143

Symmetry codes: (i) 
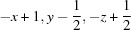
; (ii) 
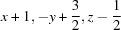
.

**Fig. 1.**
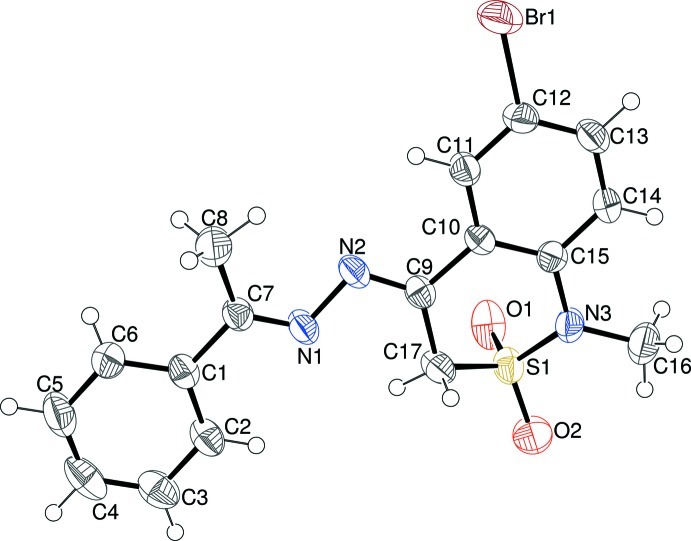
The molecular structure of (I), showing displacement ellipsoids at the 50% probability level.

## supplementary crystallographic information

### Comment

As part of our ongoing studies of benzothiazine derivatives (Shafiq, Zia-Ur-Rehman *et al.*, 2011), we now describe the synthesis and structure of the title compound, (I).

The dihedral angle between the C1–C6 and C10–C15 aromatic rings is 1.24 (15)° and the C7=N1—N2=C9 torsion angle is 167.7 (3)°. The conformation of the C9/C10/C15/C17/N3/S1 thiazine ring is an envelope, with the S atom displaced by -0.805 (3) Å from the mean plane of the other five atoms (r.m.s. deviation = 0.027 Å). Atom C16 is displaced from the mean plane by 0.081 (6) Å

In the crystal, C—H···O interactions (Table 1) link the molecules into C(10) chains propagating in [010]. A weak C—H···π interaction is also observed.

### Experimental

In the synthesis of title compound, 4-hydrazinylidene 6-bromo-1-methyl-3H-2?6,1-benzothiazine-2,2-dione (Shafiq, Khan *et al.*, 2011) was subjected to react with acetophenone according to literature procedure (Shafiq, Zia-Ur-Rehman *et al.*, 2011). The product obtained was then recrystallized in ethyl acetate under slow evaporation to obtain single crystals suitable for X-ray diffraction.

### Refinement

The H atoms were placed in calculated positions (C—H = 0.93–0.97 Å) and refined as riding. The methyl group was allowed to rotate, but not to tip, to best fit the electron density. The constraint *U*_iso_(H) = 1.2*U*_eq_(C) or 1.5*U*_eq_(methyl C) was applied.

### Crystal data

**Table:**

C_17_H_16_BrN_3_O_2_S	*F*(000) = 824
*M**_r_* = 406.30	*D*_x_ = 1.570 Mg m^−^^3^
Monoclinic, *P*2_1_/*c*	Mo *K*α radiation, λ = 0.71073 Å
Hall symbol: -P 2ybc	Cell parameters from 305 reflections
*a* = 16.4369 (13) Å	θ = 3.2–23.6°
*b* = 6.5400 (5) Å	µ = 2.53 mm^−^^1^
*c* = 16.5025 (17) Å	*T* = 296 K
β = 104.312 (4)°	Block, yellow
*V* = 1718.9 (3) Å^3^	0.34 × 0.22 × 0.20 mm
*Z* = 4	

### Data collection

**Table:**

Bruker APEXII CCD diffractometer	3213 independent reflections
Radiation source: fine-focus sealed tube	2256 reflections with *I* > 2σ(*I*)
Graphite monochromator	*R*_int_ = 0.026
ω scans	θ_max_ = 26.0°, θ_min_ = 1.3°
Absorption correction: multi-scan (*SADABS*; Bruker, 2007)	*h* = −20→17
*T*_min_ = 0.518, *T*_max_ = 0.603	*k* = −8→6
7358 measured reflections	*l* = −19→20

### Refinement

**Table:**

Refinement on *F*^2^	Primary atom site location: structure-invariant direct methods
Least-squares matrix: full	Secondary atom site location: difference Fourier map
*R*[*F*^2^ > 2σ(*F*^2^)] = 0.037	Hydrogen site location: inferred from neighbouring sites
*wR*(*F*^2^) = 0.082	H-atom parameters constrained
*S* = 1.02	*w* = 1/[σ^2^(*F*_o_^2^) + (0.0286*P*)^2^ + 0.9478*P*] where *P* = (*F*_o_^2^ + 2*F*_c_^2^)/3
3213 reflections	(Δ/σ)_max_ = 0.001
219 parameters	Δρ_max_ = 0.56 e Å^−^^3^
0 restraints	Δρ_min_ = −0.51 e Å^−^^3^

### Special details

**Table:**

Geometry. All e.s.d.'s (except the e.s.d. in the dihedral angle between two l.s. planes) are estimated using the full covariance matrix. The cell e.s.d.'s are taken into account individually in the estimation of e.s.d.'s in distances, angles and torsion angles; correlations between e.s.d.'s in cell parameters are only used when they are defined by crystal symmetry. An approximate (isotropic) treatment of cell e.s.d.'s is used for estimating e.s.d.'s involving l.s. planes.
Refinement. Refinement of *F*^2^ against ALL reflections. The weighted *R*-factor *wR* and goodness of fit *S* are based on *F*^2^, conventional *R*-factors *R* are based on *F*, with *F* set to zero for negative *F*^2^. The threshold expression of *F*^2^ > σ(*F*^2^) is used only for calculating *R*-factors(gt) *etc*. and is not relevant to the choice of reflections for refinement. *R*-factors based on *F*^2^ are statistically about twice as large as those based on *F*, and *R*- factors based on ALL data will be even larger.

### Fractional atomic coordinates and isotropic or equivalent isotropic displacement parameters (Å^2^)

**Table:**

	*x*	*y*	*z*	*U*_iso_*/*U*_eq_	
Br1	0.04673 (2)	1.15885 (6)	−0.08154 (2)	0.06740 (16)	
S1	0.43842 (5)	0.75192 (12)	0.12674 (5)	0.0441 (2)	
O1	0.42875 (15)	0.6385 (3)	0.05136 (16)	0.0603 (6)	
O2	0.51929 (13)	0.7562 (4)	0.18377 (16)	0.0698 (7)	
N1	0.23859 (15)	0.4159 (4)	0.17978 (15)	0.0421 (6)	
N2	0.21392 (15)	0.5841 (4)	0.12622 (15)	0.0421 (6)	
N3	0.40793 (14)	0.9893 (3)	0.10698 (16)	0.0424 (6)	
C1	0.20276 (18)	0.1325 (4)	0.25032 (17)	0.0363 (7)	
C2	0.28613 (19)	0.1040 (5)	0.29276 (19)	0.0460 (8)	
H2	0.3263	0.1985	0.2861	0.055*	
C3	0.3104 (2)	−0.0622 (5)	0.3447 (2)	0.0553 (9)	
H3	0.3663	−0.0774	0.3735	0.066*	
C4	0.2522 (3)	−0.2049 (5)	0.3539 (2)	0.0603 (10)	
H4	0.2690	−0.3185	0.3878	0.072*	
C5	0.1696 (3)	−0.1800 (5)	0.3132 (2)	0.0636 (10)	
H5	0.1301	−0.2761	0.3201	0.076*	
C6	0.1443 (2)	−0.0116 (5)	0.2617 (2)	0.0515 (9)	
H6	0.0880	0.0047	0.2345	0.062*	
C7	0.17779 (18)	0.3127 (4)	0.19519 (18)	0.0373 (7)	
C8	0.08710 (19)	0.3642 (5)	0.1633 (2)	0.0640 (10)	
H8A	0.0817	0.4895	0.1321	0.096*	
H8B	0.0620	0.3802	0.2096	0.096*	
H8C	0.0593	0.2561	0.1277	0.096*	
C9	0.27553 (17)	0.7046 (4)	0.12489 (17)	0.0345 (7)	
C10	0.25885 (17)	0.8882 (4)	0.07144 (16)	0.0325 (7)	
C11	0.17668 (17)	0.9302 (4)	0.02748 (17)	0.0386 (7)	
H11	0.1338	0.8406	0.0310	0.046*	
C12	0.15853 (17)	1.1031 (5)	−0.02113 (18)	0.0396 (7)	
C13	0.22109 (19)	1.2375 (5)	−0.02726 (18)	0.0429 (7)	
H13	0.2082	1.3552	−0.0595	0.051*	
C14	0.30252 (19)	1.1972 (4)	0.01430 (19)	0.0426 (7)	
H14	0.3448	1.2874	0.0093	0.051*	
C15	0.32298 (17)	1.0237 (4)	0.06383 (17)	0.0338 (7)	
C16	0.4731 (2)	1.1389 (5)	0.1033 (3)	0.0671 (11)	
H16A	0.4745	1.1602	0.0460	0.101*	
H16B	0.5267	1.0888	0.1343	0.101*	
H16C	0.4610	1.2659	0.1270	0.101*	
C17	0.36293 (17)	0.6705 (5)	0.17760 (19)	0.0445 (8)	
H17A	0.3709	0.5261	0.1906	0.053*	
H17B	0.3702	0.7444	0.2299	0.053*	

### Atomic displacement parameters (Å^2^)

**Table:**

	*U*^11^	*U*^22^	*U*^33^	*U*^12^	*U*^13^	*U*^23^
Br1	0.0446 (2)	0.0784 (3)	0.0729 (3)	0.01094 (18)	0.00247 (17)	0.0328 (2)
S1	0.0363 (4)	0.0360 (5)	0.0626 (5)	0.0044 (3)	0.0170 (4)	0.0088 (4)
O1	0.0730 (16)	0.0413 (15)	0.0809 (17)	0.0003 (11)	0.0461 (13)	−0.0069 (12)
O2	0.0325 (13)	0.0724 (18)	0.0970 (19)	0.0044 (11)	0.0016 (13)	0.0260 (15)
N1	0.0462 (15)	0.0345 (15)	0.0462 (15)	0.0026 (12)	0.0127 (12)	0.0127 (12)
N2	0.0451 (15)	0.0345 (15)	0.0457 (15)	0.0039 (12)	0.0091 (12)	0.0148 (12)
N3	0.0374 (14)	0.0298 (15)	0.0572 (16)	−0.0033 (11)	0.0062 (12)	0.0031 (12)
C1	0.0480 (19)	0.0295 (18)	0.0336 (16)	−0.0005 (13)	0.0145 (14)	−0.0012 (12)
C2	0.049 (2)	0.041 (2)	0.052 (2)	0.0096 (15)	0.0204 (16)	0.0132 (15)
C3	0.057 (2)	0.059 (2)	0.053 (2)	0.0190 (18)	0.0203 (18)	0.0145 (18)
C4	0.102 (3)	0.037 (2)	0.049 (2)	0.017 (2)	0.030 (2)	0.0117 (16)
C5	0.097 (3)	0.042 (2)	0.053 (2)	−0.021 (2)	0.021 (2)	0.0062 (17)
C6	0.056 (2)	0.047 (2)	0.046 (2)	−0.0148 (16)	0.0038 (16)	0.0029 (16)
C7	0.0430 (18)	0.0295 (17)	0.0390 (17)	0.0006 (13)	0.0094 (14)	0.0023 (13)
C8	0.043 (2)	0.058 (2)	0.087 (3)	−0.0007 (16)	0.0081 (19)	0.025 (2)
C9	0.0372 (17)	0.0308 (18)	0.0380 (16)	0.0051 (13)	0.0142 (13)	0.0034 (12)
C10	0.0373 (17)	0.0290 (17)	0.0315 (15)	0.0044 (12)	0.0092 (13)	0.0028 (12)
C11	0.0382 (17)	0.0370 (18)	0.0421 (17)	0.0001 (13)	0.0126 (14)	0.0072 (14)
C12	0.0361 (17)	0.043 (2)	0.0384 (17)	0.0062 (14)	0.0071 (13)	0.0057 (14)
C13	0.053 (2)	0.0322 (18)	0.0419 (18)	0.0047 (15)	0.0085 (15)	0.0085 (14)
C14	0.0467 (19)	0.0301 (18)	0.0496 (19)	−0.0046 (13)	0.0091 (15)	0.0069 (14)
C15	0.0379 (17)	0.0296 (17)	0.0335 (16)	0.0004 (12)	0.0080 (13)	−0.0032 (12)
C16	0.051 (2)	0.042 (2)	0.099 (3)	−0.0104 (16)	0.001 (2)	0.0071 (19)
C17	0.0399 (18)	0.048 (2)	0.0470 (19)	0.0082 (14)	0.0144 (15)	0.0176 (15)

### Geometric parameters (Å, º)

**Table:**

Br1—C12	1.897 (3)	C6—H6	0.9300
S1—O1	1.423 (2)	C7—C8	1.491 (4)
S1—O2	1.427 (2)	C8—H8A	0.9600
S1—N3	1.639 (2)	C8—H8B	0.9600
S1—C17	1.744 (3)	C8—H8C	0.9600
N1—C7	1.282 (4)	C9—C10	1.475 (4)
N1—N2	1.407 (3)	C9—C17	1.501 (4)
N2—C9	1.288 (3)	C10—C11	1.393 (4)
N3—C15	1.420 (3)	C10—C15	1.407 (4)
N3—C16	1.464 (4)	C11—C12	1.376 (4)
C1—C2	1.389 (4)	C11—H11	0.9300
C1—C6	1.391 (4)	C12—C13	1.375 (4)
C1—C7	1.484 (4)	C13—C14	1.370 (4)
C2—C3	1.381 (4)	C13—H13	0.9300
C2—H2	0.9300	C14—C15	1.390 (4)
C3—C4	1.371 (5)	C14—H14	0.9300
C3—H3	0.9300	C16—H16A	0.9600
C4—C5	1.369 (5)	C16—H16B	0.9600
C4—H4	0.9300	C16—H16C	0.9600
C5—C6	1.391 (5)	C17—H17A	0.9700
C5—H5	0.9300	C17—H17B	0.9700
			
O1—S1—O2	118.12 (15)	H8A—C8—H8C	109.5
O1—S1—N3	110.94 (14)	H8B—C8—H8C	109.5
O2—S1—N3	107.57 (13)	N2—C9—C10	118.5 (3)
O1—S1—C17	108.81 (15)	N2—C9—C17	122.9 (3)
O2—S1—C17	110.25 (15)	C10—C9—C17	118.5 (2)
N3—S1—C17	99.55 (13)	C11—C10—C15	118.7 (2)
C7—N1—N2	114.7 (2)	C11—C10—C9	119.0 (2)
C9—N2—N1	112.6 (2)	C15—C10—C9	122.3 (2)
C15—N3—C16	120.8 (2)	C12—C11—C10	120.5 (3)
C15—N3—S1	117.68 (18)	C12—C11—H11	119.7
C16—N3—S1	116.8 (2)	C10—C11—H11	119.7
C2—C1—C6	118.0 (3)	C13—C12—C11	120.7 (3)
C2—C1—C7	120.3 (3)	C13—C12—Br1	118.9 (2)
C6—C1—C7	121.7 (3)	C11—C12—Br1	120.4 (2)
C3—C2—C1	121.1 (3)	C14—C13—C12	119.7 (3)
C3—C2—H2	119.5	C14—C13—H13	120.1
C1—C2—H2	119.5	C12—C13—H13	120.1
C4—C3—C2	120.2 (3)	C13—C14—C15	121.0 (3)
C4—C3—H3	119.9	C13—C14—H14	119.5
C2—C3—H3	119.9	C15—C14—H14	119.5
C5—C4—C3	120.0 (3)	C14—C15—C10	119.3 (3)
C5—C4—H4	120.0	C14—C15—N3	119.3 (2)
C3—C4—H4	120.0	C10—C15—N3	121.4 (2)
C4—C5—C6	120.3 (3)	N3—C16—H16A	109.5
C4—C5—H5	119.9	N3—C16—H16B	109.5
C6—C5—H5	119.9	H16A—C16—H16B	109.5
C5—C6—C1	120.5 (3)	N3—C16—H16C	109.5
C5—C6—H6	119.8	H16A—C16—H16C	109.5
C1—C6—H6	119.8	H16B—C16—H16C	109.5
N1—C7—C1	115.4 (3)	C9—C17—S1	111.6 (2)
N1—C7—C8	124.9 (3)	C9—C17—H17A	109.3
C1—C7—C8	119.8 (3)	S1—C17—H17A	109.3
C7—C8—H8A	109.5	C9—C17—H17B	109.3
C7—C8—H8B	109.5	S1—C17—H17B	109.3
H8A—C8—H8B	109.5	H17A—C17—H17B	108.0
C7—C8—H8C	109.5		
			
C7—N1—N2—C9	167.7 (3)	N2—C9—C10—C15	−177.5 (3)
O1—S1—N3—C15	60.7 (2)	C17—C9—C10—C15	4.6 (4)
O2—S1—N3—C15	−168.7 (2)	C15—C10—C11—C12	−1.3 (4)
C17—S1—N3—C15	−53.8 (2)	C9—C10—C11—C12	178.2 (3)
O1—S1—N3—C16	−95.2 (3)	C10—C11—C12—C13	0.1 (4)
O2—S1—N3—C16	35.4 (3)	C10—C11—C12—Br1	179.5 (2)
C17—S1—N3—C16	150.3 (3)	C11—C12—C13—C14	1.1 (5)
C6—C1—C2—C3	0.0 (4)	Br1—C12—C13—C14	−178.4 (2)
C7—C1—C2—C3	179.6 (3)	C12—C13—C14—C15	−1.0 (5)
C1—C2—C3—C4	1.3 (5)	C13—C14—C15—C10	−0.2 (4)
C2—C3—C4—C5	−1.7 (5)	C13—C14—C15—N3	−179.0 (3)
C3—C4—C5—C6	0.8 (5)	C11—C10—C15—C14	1.3 (4)
C4—C5—C6—C1	0.5 (5)	C9—C10—C15—C14	−178.1 (3)
C2—C1—C6—C5	−0.8 (5)	C11—C10—C15—N3	−180.0 (2)
C7—C1—C6—C5	179.5 (3)	C9—C10—C15—N3	0.6 (4)
N2—N1—C7—C1	178.9 (2)	C16—N3—C15—C14	3.1 (4)
N2—N1—C7—C8	−2.3 (4)	S1—N3—C15—C14	−151.7 (2)
C2—C1—C7—N1	10.8 (4)	C16—N3—C15—C10	−175.6 (3)
C6—C1—C7—N1	−169.5 (3)	S1—N3—C15—C10	29.5 (3)
C2—C1—C7—C8	−168.0 (3)	N2—C9—C17—S1	146.5 (3)
C6—C1—C7—C8	11.6 (4)	C10—C9—C17—S1	−35.7 (3)
N1—N2—C9—C10	−179.9 (2)	O1—S1—C17—C9	−60.6 (2)
N1—N2—C9—C17	−2.1 (4)	O2—S1—C17—C9	168.4 (2)
N2—C9—C10—C11	3.1 (4)	N3—S1—C17—C9	55.5 (2)
C17—C9—C10—C11	−174.8 (3)		

### Hydrogen-bond geometry (Å, º)

**Table:**

*D*—H···*A*	*D*—H	H···*A*	*D*···*A*	*D*—H···*A*
C2—H2···O2^i^	0.93	2.49	3.280 (4)	143
C13—H13···*Cg*2^ii^	0.93	2.65	3.445 (3)	143

Symmetry codes: (i) −*x*+1, *y*−1/2, −*z*+1/2; (ii) *x*+1, −*y*+3/2, *z*−1/2.
